# Dynamic Changes in Intestinal Microorganisms and Hematological Indices in Giraffes of Different Ages, and the Effect of Diarrhea on Intestinal Microbiota

**DOI:** 10.3390/ani14233379

**Published:** 2024-11-24

**Authors:** Baisheng Yu, Hangfan Li, Qiong Chen, Chuang Yang, Yongqing Guo, Baoli Sun

**Affiliations:** College of Animal Science, South China Agriculture University, Guangzhou 510642, China; ybs18022349501@163.com (B.Y.); 20223139043@stu.scau.edu.cn (H.L.); 13253749296@163.com (Q.C.); ychuang1139@163.com (C.Y.); yongqing@scau.deu.cn (Y.G.)

**Keywords:** giraffe (camelopardalis), intestinal microbial structure, dominant microbes, diarrhea, hematology

## Abstract

This study employed high-throughput sequencing to explore bacterial diversity and hematological variations across different age groups of giraffes, as well as the impact of diarrhea on their intestinal microbiota. Additionally, the correlation between intestinal flora and hematological indices was examined for the first time. Firmicutes, Bacteroides, and Proteobacteria were the predominant bacterial groups in the giraffe’s intestinal flora. The α-diversity analysis indicated significant variations in microbial diversity among giraffes of varying ages (*p* < 0.05). Furthermore, giraffes suffering from diarrhea exhibited significant alterations in the abundance of Proteobacteria and Actinobacteriota at the phylum level (*p* < 0.05). At the genus level, *Rikenellaceae_RC9_gut_group*, *Monoglobus*, and *Prevotellaceae_UCG-004* had significant differences compared to healthy counterparts (*p* < 0.05). Hematological parameters such as lymphocyte count (Lym), red blood cell count (RBC), mean corpuscular volume (MCV), mean corpuscular hemoglobin (MCH), mean corpuscular hemoglobin concentration (MCHC), platelet count (PLT), and plateletcrit (PCT) varied significantly across different age groups (*p* < 0.05). A substantial correlation was observed between the intestinal microbiome composition and hematological parameters (*p* < 0.05). In conclusion, this study highlights significant differences in both the intestinal microbiome composition and hematological indices among giraffes of different ages. Diarrhea was found to significantly alter the abundance and composition of the intestinal microbial community. These insights provide a valuable theoretical foundation for the microbiological and hematological aspects of giraffe health management and breeding practices.

## Simple Summary

Giraffes are the tallest terrestrial mammals in the world, with complex gastrointestinal microbiota, and are one of the most popular exhibits in zoos. However, there is relatively little research on the intestinal microbiota and hematological indicators of giraffes. Moreover, giraffes in captivity may face challenges such as energy malnutrition, intestinal parasitic diseases, and chronic emaciation, and some serious ones may even lead to death-acute death syndrome. Therefore, how to scientifically keep giraffes healthy in captivity remains a challenge. This study uses high-throughput sequencing technology to explore the diversity of bacteria and hematological differences among giraffes of different ages, as well as the impact of diarrhea on their intestinal microbiota. By understanding the physiological characteristics of this rare species, it provides a theoretical basis for its future protection and health management.

## 1. Introduction

The intestinal microbiome represents the largest symbiotic ecosystem within its host, playing a crucial role in maintaining intestinal equilibrium [1]. It significantly influences the host’s intestinal health, digestion, absorption of nutrients, and immune function [2]. During early animal development, initial microbial colonization is transmitted from mother to offspring, with bacteria selectively conveyed to the newborn’s intestines via the intestinal–mammary route, which is vital for neonatal development [3]. An imbalance in the intestinal microflora can lead to the proliferation and colonization of opportunistic pathogens, triggering inflammatory responses that severely impact animal health, such as diarrhea [4].

Giraffes (*Giraffa camelopardalis*), primarily inhabiting the African continent, stand as the tallest terrestrial mammals and harbor a complex gastrointestinal microbial ecosystem [5]. With nine recognized subspecies, giraffe populations are currently dwindling, classifying them as “Vulnerable” on the IUCN Red List [6,7]. As popular zoo exhibits, ensuring the welfare of captive giraffes is vital due to their specialized dietary and housing requirements [8]. Common issues in captive giraffes, such as diarrhea and malnutrition, are often linked to an imbalance in intestinal microorganisms [9]. Additionally, giraffes can be infected with pathogenic bacteria, leading to infectious diseases [10,11].

While research on the microbial community of ruminants has often focused on domestic members of the Bovidae family, less is known about the microbiome of wild ruminants. Recent studies have analyzed the role of intestinal flora in the development of wild animals. Wang et al. [12] elucidated the evolution of the intestinal microbial structure in the giant panda and its relationship with cellulase activity. Jiang et al. [13] examined the composition, diversity, and gender differences of the intestinal flora in captive African penguins. Neumann et al. [14] identified potential correlations between bacterial communities in the rumen and feces. Understanding the composition and diversity of intestinal microorganisms in giraffes aids in comprehending the physiological characteristics of this rare species and is crucial for their conservation and management. Furthermore, as nutrients produced by intestinal microorganisms are absorbed into the bloodstream during ruminant digestion, routine blood and biochemical indices are commonly used for evaluating animal health and dietary nutrition [15]. Debra et al. [16] further evaluated the health status of captive giraffes by comparing the serum chemical values between captive and free-range giraffes.

However, there are few reports about the dynamic changes of the intestinal microorganism structure in giraffes at different stages (cub, sub-adult, and adult) and the succession law of intestinal microorganisms throughout their growth and development. The changes in the blood indices of captive giraffes at different development stages are also unclear. Moreover, the relationship between giraffe diarrhea and intestinal microorganisms remains unresolved. Therefore, this study utilized high-throughput sequencing technology to sequence the intestinal microorganisms of captive giraffes, analyzing compositional differences and diversity at various ages. By collecting blood and analyzing routine and biochemical parameters, this study aims to provide valuable theoretical insights for giraffe condition evaluation and health management.

## 2. Materials and Methods

### 2.1. Research Object

In this study, fecal samples were collected from 18 healthy captive giraffe (cubs: 0–1 years old giraffes, sub-adults: 1–3 years old giraffes, and adults: 10–12 years old giraffes) in different stages of development and 6 diarrhea giraffes that were kept in Qingyuan Chimelong Safari Park. Giraffe recipes and basic information can be found in the Supplementary Materials. The fecal samples were collected in April 2023, and the blood samples were collected by a professional veterinarian in the early morning. The specific process of blood collection is as follows: the veterinarian will stand on the platform of the treatment cage so that the veterinarian can take blood from the giraffe’s jugular venipuncture. During the sampling period, except for the 6 diarrhea giraffes, the other sample giraffes were all in good condition with no abnormalities such as illness or abnormal excretion. In addition, giraffe cubs feed mainly on breast milk, supplemented by alfalfa hay and fresh leaves, and occasionally eat some concentrate. Sub-adult giraffes can eat approximately 10 kg alfalfa hay and 3 kg concentrate per day. Adult giraffes can eat approximately 15 kg alfalfa hay and 3 kg concentrate per day.

### 2.2. Sample Collection and Storage

Fresh dung from healthy giraffes was collected every morning during the last three days of April 2023 before the animals consumed their first meal of the day. What needs to be explained is that, due to the requirement of management of the safari park, we could only collect 6 feces samples every day. For giraffes experiencing diarrhea, fecal samples were collected in real time within the veterinary treatment cage. When a giraffe defecated, a sample was immediately collected under the supervision of senior breeders, ensuring minimal contamination. Each feces sample was collected by a sterile spoon and immediately placed in a cryogenic storage tube (Thermo Fisher Scientific, Guangzhou, China) to avoid environment pollution and stored in a liquid nitrogen tank. After returning to the laboratory, the sample in liquid nitrogen was transferred to the refrigerator at −80 °C for storage. A total of 24 blood samples were collected from 18 healthy giraffes and 6 diarrhea giraffes. Giraffes’ blood was taken from the giraffes’ jugular venipuncture by a professional veterinarian and collected in an anticoagulant blood collection tube with EDTA and non-anticoagulant vacuum blood collection tube, then were centrifuged to collect plasma and serum after clotting (3000× *g* for 15 min at room temperature). Then, the blood samples were stored in the refrigerator at 80 °C in Chimelong Veterinary Hospital.

### 2.3. Analysis of the Intestinal Microbial Diversity of Giraffes

Following thawing of the sample, microbial DNA was extracted using a HiPure Stool DNA Kit (Magen, Guangzhou, China) according to the manufacturer’s protocols. The 16S rDNA target region of the ribosomal RNA gene was amplified by PCR (95 °C for 5 min, followed by 30 cycles at 95 °C for 1 min, 60 °C for 1 min, 72 °C for 1 min, and a final extension at 72 °C for 7 min) using the primer name (341F and 806R) and primer sequence (CCTACGGGNGGCWGCAG and GGACTACHVGGGTATCTAAT). We used a 50 µL mixture containing 10 µL of 5 × Q5@ Reaction Buffer, 10 µL of 5 × Q5@ High GC Enhancer, 1.5 µL of 2.5 mM dNTPs, 1.5 µL of each primer (10 µM), 0.2 µL of Q5@ High-Fidelity DNA Polymerase, and 50 ng of template DNA. Related PCR reagents were from New England Biolabs, USA. Amplicons were evaluated with 2% agarose gels and purified using the AMPure XP Beads (Beckman, CA, USA), according to the manufacturer’s instructions. Sequencing libraries were generated using an Illumina DNA Prep Kit (Illumina, CA, USA) following the manufacturer’s recommendation. The library quality was assessed with an ABI StepOnePlus Real-Time PCR System (Life Technologies, Foster City, CA, USA). At the end, 2 × 250 bp paired-end reads were generated by sequencing on the Novaseq 6000 platform. The raw reads were deposited into the NCBI Sequence Read Archive (SRA) database.

### 2.4. Analysis of the Blood Routine and Blood Biochemistry in Giraffes

Blood routine and biochemistry were conducted by using a BC-2800 automatic blood cell analyzer and IDEXX Catalyst One automatic biochemical analyzer (Mindray Medical India Pvt. Ltd., Gurugram, India), respectively, in Qingyuan Chimelong Veterinary Hospital.

Blood routine tests included WBC: white blood cell count; Neu#: neutrophil; Lym#: lymphocyte (Lym); Mon#: monocyte; Eos#: eosinophil; Bas#: basophils; RBC: red blood cell count; HGB: hemoglobin concentration; HCT: hematocrit; MCV: mean red hematocrit; MCH: mean hemoglobin content; MCHC: mean hemoglobin concentration; RDW-CV: red cell distribution width-coefficient of variation; RDW-SD: red cell distribution width-standard deviation; PLT: platelet number; MPV: mean platelet volume; PDW: platelet distribution width; PCT: platelet hematocrit.

Blood biochemistry tests included ALT: alanine aminotransferase activity; AST: aspartate aminotransferase; ALP: alkaline phosphatase; GGT: glutamyl transferase activity; T-bil: total bilirubin; D-bil: direct bilirubin; TBA: total bile acid; TP: total protein; ALB: albumin; GLB: globulin; CHE: choline esterase; ADA: adenosine deaminase; CREA: creatinine; Glu: blood glucose; TG: triglyceride; TC: total cholesterol; Ca: calcium; P: inorganic phosphorus; Mg: magnesium; Fe: serum iron; CO_2_: carbondioxide; AMY: pancreatic amylase activity; CK: creatine kinase activity; CK-MB: creatine kinase isoenzyme; LDH: lactic acid dehydrogenase activity; CRP: C-reactive protein; UREA: urea.

### 2.5. Statistics Analysis

The original data produced from high-throughput sequencing were performed by quality screening using the DADA2 R package (version 1.14) to achieve reliable results in the subsequent bioinformatics analysis. Based on the UCHIME algorithm, Chimera sequences were identified and deleted to output denoised and chimera-free ASV sequences and their abundances. The representative ASV sequences were classified into organisms by a naive Bayesian model using a RDP classifier (version 2.2) based on the UNITE database (version 8.3), with the confidence threshold value of 0.8. The stacked bar plot of the community composition was visualized in the R project ggplot2 package (version 2.2.1). A network of correlation coefficient were generated using the igraph package (version 1.1.2) in R project. Between-groups Venn analysis was performed in R project Venn Diagram package (version 1.6.16), and UpSet plot was performed in R project UpSet R package (version 1.3.3) to identify unique and common species or ASVs. Species comparisons among groups were computed by Tukey’s HSD test and the Kruskal–Wallis H test in the R project Vegan package (version 2.5.3). Biomarker features in each group were screened by LEfSe software (version 1.0), randomforest package (version 4.6.12) in R project, pROC package (version 1.10.0) in R project, and labdsv package (version2.0-1) in R project. The Chao1, Shannon, Simpson, and Pielou’s Evenness indices were calculated in QIIME (version 1.9.1). Rank abundance curves were plotted in R project ggplot2 package (version 2.2.1). The Bray–Curtis distance matrix was calculated in R project Vegan package (version 2.5.3). Multivariate statistical techniques, including PCoA (principal coordinates analysis) of Bray–Curtis distances were generated in R project Vegan package (version 2.5.3) and plotted in R project ggplot2 package (version 2.2.1). Statistic analysis of Adonis (also called PERMANOVA) was calculated in R project Vegan package (version 2.5.3). The KEGG pathway analysis of the OTUs/ASV was inferred using PICRUSt (version 2.1.4). Statistical analysis of the data was performed using R project Vegan package (version 2.5.3) and GraphPad Prism (version 9.0c). *p*-values < 0.05 were considered statistically significant, and the values were presented as means ± SD.

## 3. Results

### 3.1. Composition of the Bacterial Community in Giraffes at Different Development and Diarrhea Stages

In this microbial experiment of giraffe feces, a totally of 24 feces samples from different ages of giraffes and diarrhea giraffes were subjected to amplicon sequencing, and a total of 3,075,578 original sequences were acquired from the V3/4 regions via Illumina high-throughput sequencing technology. After quality control, denoising, and assembly, there were 2,139,311 qualified sequences obtained with an average of length of 412 bp in total. According to the output result of DADA2 software, 7177 ASVs were recognized on average based on 100% nucleotide sequence similarity. Both species accumulation and rarefaction curves per sample were relatively flat and showed a tendency to saturate the characteristics, indicating that nearly all the bacterial species were identified in the fecal samples of the giraffes.

**Figure 1:**
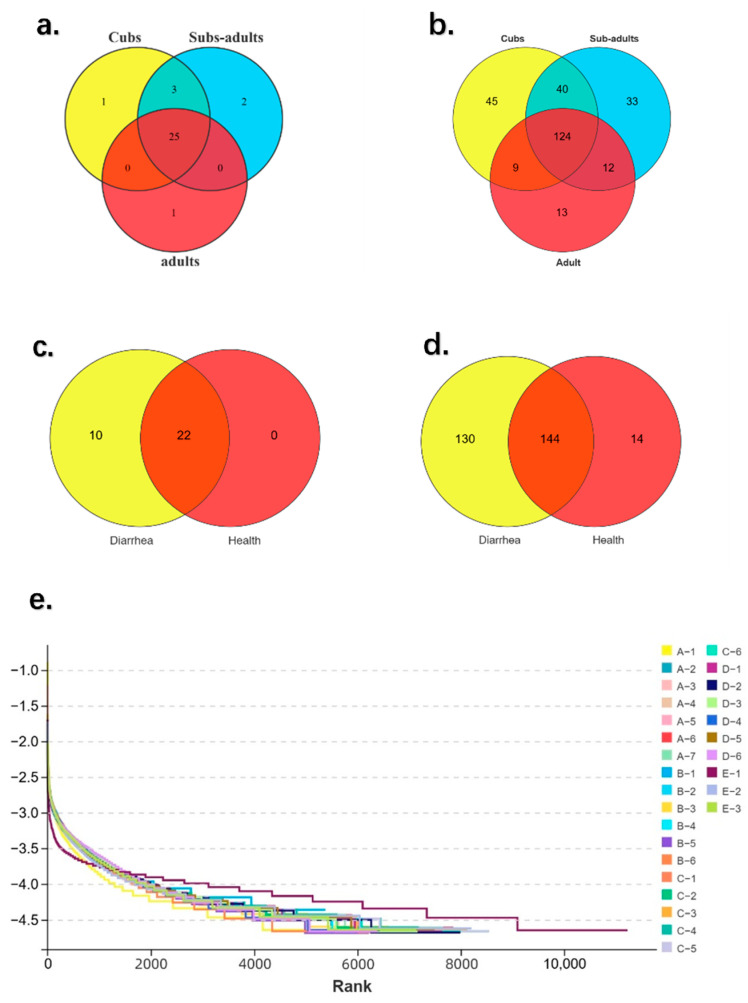
Venn diagrams and sample feasibility analysis between both groups. (**a**,**b**) A Venn diagram illustrating the distribution of ASVs in the feces of giraffes with different ages, stratified at both the phylum and genus levels. (**c**,**d**) A Venn diagram illustrating the distribution of ASVs in the feces of giraffes with diarrhea and healthy giraffes, stratified at both the phylum and genus levels. (**e**) Rank abundance curve.

According to Figure 1, group cubs, group sub-adults, and group adults share 25 ASVs, and have 1, 2, and 1 unique ASVs. Respectively, at the phylum level. Meanwhile, at the genus level, it changed to share 124 ASVs and have 45, 33, and 13 unique ASVs, respectively. However, there is a big difference between the diarrhea group and the healthy group. They share 22 and 144 ASVs at the phylum level and the genus level, respectively. Among them, the diarrhea group has 10 unique ASVs at the phylum level and 130 ASVs at the genus level, which shows the difference between diarrhea and healthy.

**Figure 2:**
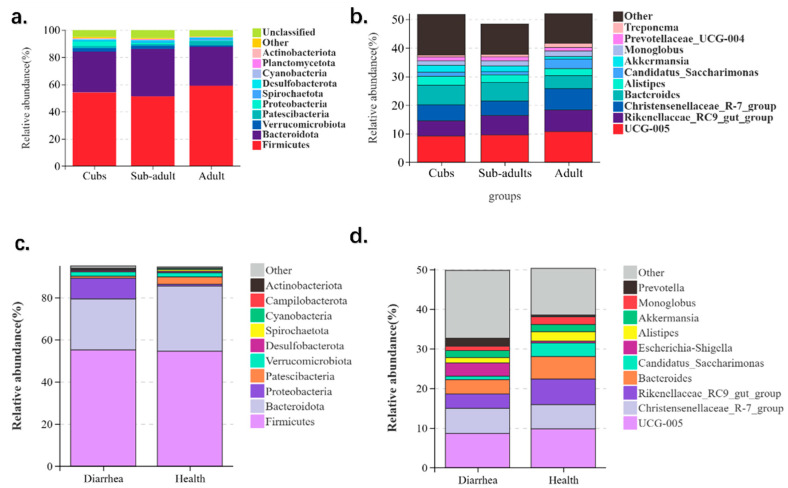
(**a**,**b**) Bar diagram of intestinal dominant bacteria abundance in the phylum and genera of different developmental stages for giraffes. (**c**,**d**) Bar diagram of intestinal dominant bacteria abundance in the phylum and genera of diarrhea and healthy giraffes.

Figure 2 shows the microbial compositions of different age groups of giraffes, the predominant genera at the phylum level of bacteria in the intestines of giraffe cubs during its period were Firmicutes (54.122%), Bacteroidota (30.102%), Proteobacteria (3.376%), Verrucomicrobiota (2.667%), and Patescibacteria (1.416%). The most dominant bacterial genera in the intestine during the sub-adult period were Firmicutes (51.189%), Bacteroidota (35.08%), Verrucomicrobiota (2.258%), Proteobacteria (1.088%), and Patescibacteria (1.067%). On the other hand, the dominant bacterial genera in the intestines during adulthood were Firmicutes (59.115%), Bacteroidota (28.55%), Spirochaetota (1.504%), Patescibacteria (3.247%), and Verrucomicrobiota (1.083%). Among of them, the abundance of Firmicutes, Desulfobacterota, and Cyanobacteria in group adults were all significantly higher than those in group sub-adults (*p* < 0.05).

**Figure 3:**
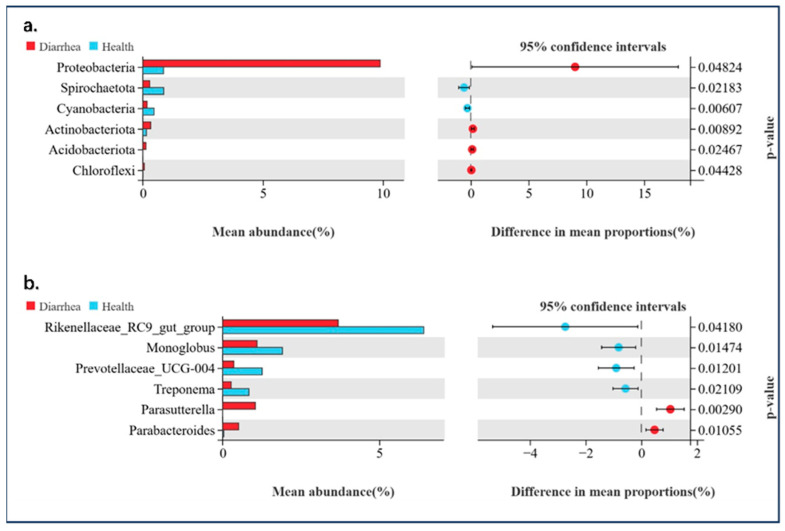
(**a**,**b**) Significant shifts in gut microbial abundance at the phylum and genus levels in giraffes.

At the genus level of bacteria, *UCG-005*, *Christensenellaceae_R-7_group*, *Rikenellaceae_RC9_gut_group*, *Bacteroides*, and *Alistipes* are the predominant genera in the intestines of giraffes in groups cubs, sub-adults, and adults (Figure 3). Among those, the abundance of *Rikenellaceae_RC9_gut_group* in group adult is significantly higher than that in group sub-adults and group cubs (*p* < 0.05).

To dissect the shifts in microbial feces between diarrhea and healthy giraffes, we also sequenced 16S microorganisms in the feces of these two groups. As Figure 3 shows, at the phylum level, the predominant bacterial genera were Firmicutes, Bacteroidota, Proteobacteria, Verrucomicrobiota, Patescibacteria, Spirochaetota, Planctomycetota, Desulfobacterota, Actinobacteriota, and Cyanobacteria. Moreover, the abundance of Proteobacteria, Spirochaetota, Actinobacteriota, and Cyanobacteria had a significant difference (*p* < 0.05). On the other hand, *UCG-005*, *Christensenellaceae_R-7_group*, *Rikenellaceae_RC9_gut_group*, *Bacteroides*, *Alistipes*, *Akkermansia*, *Monoglobus*, *Candidatus_Saccharimonas*, *Prevotella*, and *Prevotellaceae_UCG-004* are the predominant genera at the genus level, and the abundance of *Rikenellaceae_RC9_gut_group*, *Monoglobus*, and *Prevotellaceae_UCG-004* are significantly different (*p* < 0.05) between diarrhea and healthy giraffes (Figure 3).

### 3.2. Bacterial Diversity of Giraffes at Different Development and Diarrhea Stages

#### 3.2.1. Intestinal Microbial of Giraffes on α-Diversity at Different Developmental Stages

**Figure 4:**
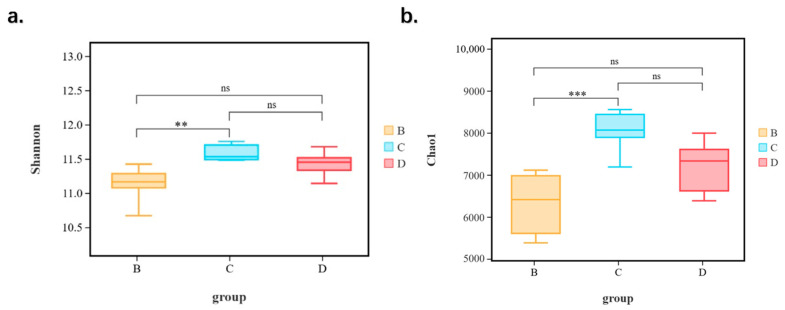
Alpha diversity (Chao1 and Shannon) indices of intestinal microbiota between different ages of giraffes. (B, C, and D represent cubs, sub-adults, and adults, respectively). (**a**) Box diagram of shannon index between different groups. (**b**) Box diagram of Chao1 index between different groups. ** represents *p* < 0.01; *** represents *p* < 0.001; ns represents not significant.

Alpha diversity reflects the richness, diversity, and evenness of microbiota in a host. According to the Good’s coverage index, which can assess the coverage of sequencing data, each sample was above 0.99, indicating that the sequencing data for all samples were reliable. The Chao1 indices can reflect the richness of the microbial structure. Figure 4 shows that the Chao1 indices in group sub-adults are significantly higher than those in group cubs (*p* < 0.01), which means that the richness of the intestinal bacterial flora changes during the development of giraffes. On the other hand, the Shannon and Simpson indices can evaluate the diversity of intestinal microbiota. Figure 4 indicates that there was a significant difference between group cubs, sub-adults, and adults (*p* < 0.05). Among that, the Shannon index in group sub-adults is significantly higher than group cubs (*p* < 0.05), and the Simpson index in group adults is also significantly higher than group cubs (*p* < 0.05), which indicate that the age factor has a direct influence on the diversity of intestinal microbiota in giraffes. However, all alpha diversity indices showed no significant differences between the diarrhea and healthy groups, suggesting that the diarrhea factor had no direct influence on the diversity of intestinal microbiota in giraffes.

#### 3.2.2. Intestinal Microbial of Giraffes on β-Diversity at Different Developmental Stages

**Figure 5:**
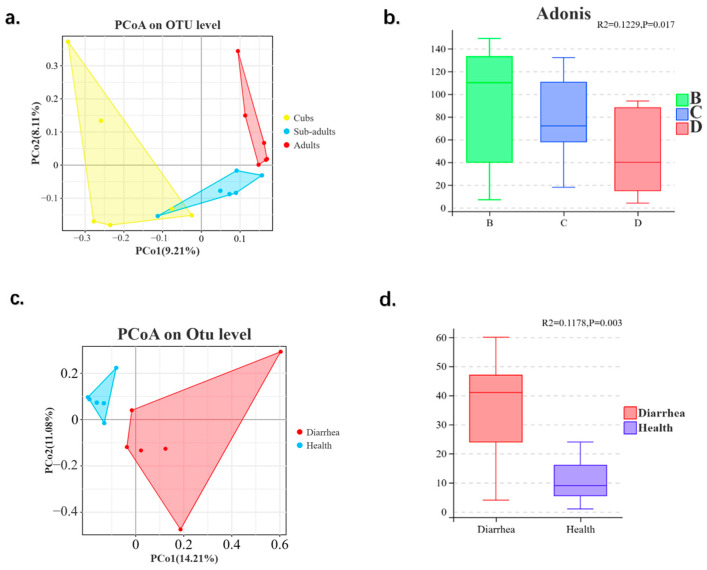
(**a**,**c**) PCoA analysis of intestinal bacterial communities in giraffes. (**b**,**d**) Box chart of Adonis difference analysis. (B, C, and D represent cubs, sub-adults, and adults, respectively).

Beta diversity can reflect the similarity of microbial species in different groups. According to Figure 5, PCoA revealed that the intestinal microbiota in different age groups was distinctly separated. Then, we used analysis of similarities (ANOSIM) to further elucidate the differences between intergroup and intragroup. The results of ANOSIM showed that R = 0.1229 (*p* < 0.01), which indicated that the difference in beta diversity between group cubs, group sub-adults, and adults was significantly greater than the difference within the groups. Similarly, the intestinal microbiota in group diarrhea was distinctly separated from group healthy, according to Figure 5. The results of ANOSIM showed that R = 0.1178 (*p* < 0.01), which also indicated the differences in beta diversity between group diarrhea and group healthy.

### 3.3. Giraffes’ Development and Diarrhea on the Alteration and Composition of Intestinal Bacteria

**Figure 6:**
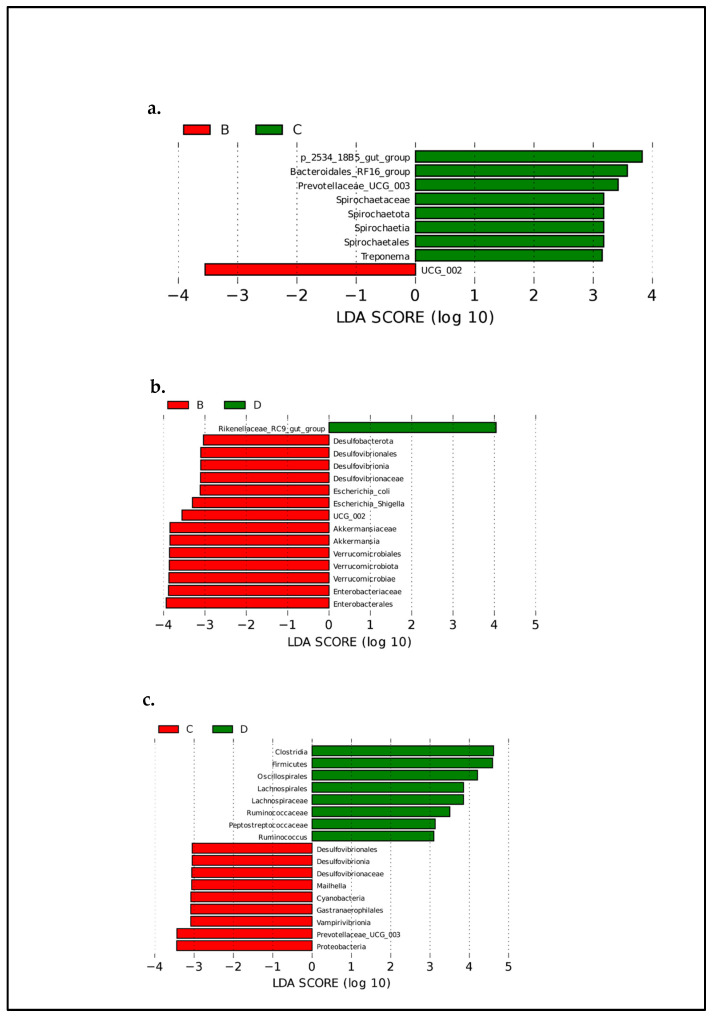
(**a**–**c**) LDA scores displaying the distinct bacterial differences between different ages of giraffes. LDA scores > 3.0 were considered statistically significant. (B, C, and D represent cubs, sub-adults, and adults, respectively; cubs: 0–1 years old healthy giraffes; sub-adults: 1–3 years old healthy giraffes; adults: 10–12-year-old healthy giraffes).

**Figure 7:**
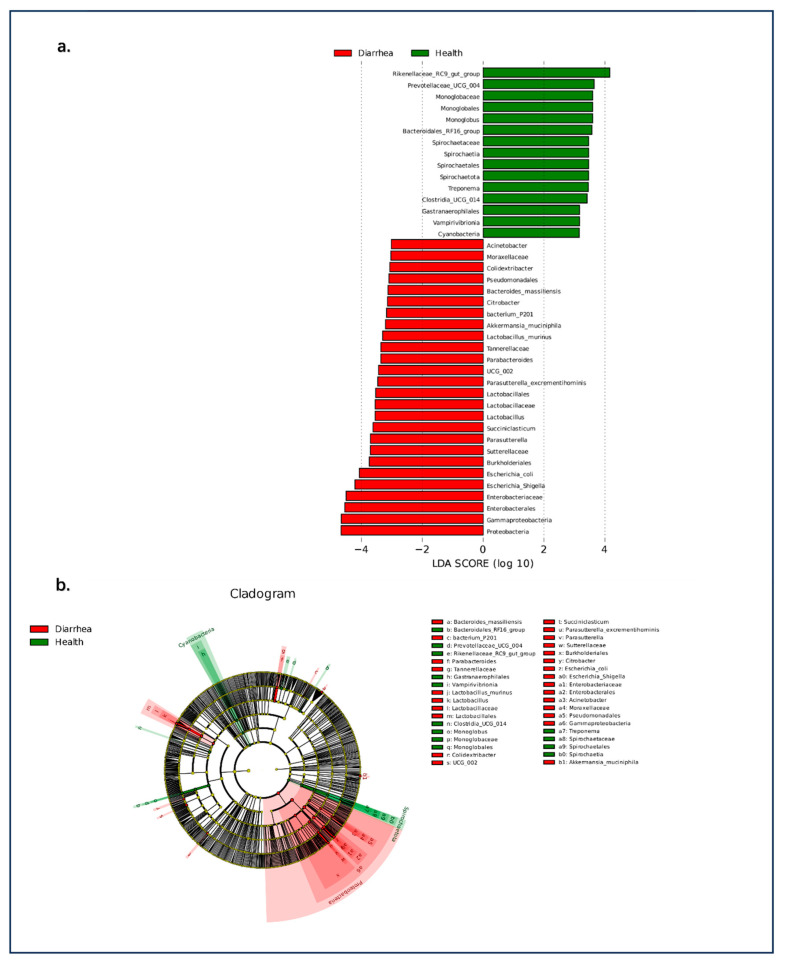
(**a**) LDA scores displaying the distinct bacterial differences between diarrhea and healthy. LDA scores > 3.0 were considered statistically significant. (**b**) Cladogram revealing phylogenetic distribution of the gut bacterial community associated with diarrhea and healthy giraffes.

LEfSe analysis (LDA effect size) was used to compare two groups, and LDA (linear discriminant analysis) (a supervised classification method) can be used to reduce the dimension and evaluate the influence of significantly different species (LDA score). Significant taxonomic differences in bacteria (the linear discriminant analysis threshold is 3) are shown in the evolutionary diagram of the LEfSe analysis (Figure 6), suggesting there are age-related differences in the relative abundances of intestinal microbiota in giraffes. Compared to the other groups, cub giraffes had higher abundances of *Desulfobacterota*, *Escherichia*, *Akkermansia*, *Verrucomicrobiota*, and *Enterobacteriaceae*. It may indicate that these bacteria, which easily cause intestinal flora disorder, are more likely to colonize in the intestines of young giraffes. While compared to sub-adult giraffes, *Clostridia*, *Firmicutes, Oscillospirales, Lachnospirales, Peptostreptococcaceae*, and *Ruminococcaceae* were the higher abundance microbiota in adult giraffes, which may indicate that these bacteria related to the stability of intestinal flora usually play an important role in the intestinal flora of older giraffes. On the other hand, linear discriminant analysis effect size analysis coupled with linear discriminant analysis was performed to identify the specific bacteria associated with diarrhea (Figure 7). Compared to group healthy, giraffes had a lower abundance of *Proteobacteria, Gammaproteobacteria, Enterobacterales, Enterobacteriaceae*, and *Escherichia-Shigella* in group diarrhea. Meanwhile, the healthy group exhibited higher abundances of *Rikenellaceae_RC9_gut_group* and *Prevotellaceae_UCG-004*.

### 3.4. Results of the Blood Indices

The serum chemistry value and routine blood test indices are typical analytical tools to further assess the health status of captive giraffes. According to Table 1, the following routine blood test indices, such as lymphocyte (Lym), red blood cell count (RBC), mean red hematocrit (MCV), mean hemoglobin content (MCH), mean hemoglobin concentration (MCHC), platelet number (PLT), and platelet hematocrit (PCT), all had significant differences between different age groups. Among them, the indices of Lym, RBC, MCHC, PCT, and PLT in group adults were significantly lower than those in group cubs and group sub-adults. On the contrary, the indices of MCV and MCH were significantly higher than group cubs and group sub-adults. Interestingly, the index of Mon in group sub-adults was significantly higher than the other groups.

Table 2 shows the serum chemistry values of giraffes at different developmental stages. Among those, the values of aspartate aminotransferase (AST), alkaline phosphatase (ALP), adenosine deaminase (ADA), creatinine (CREA), blood glucose (Glu), triglyceride (TG), total cholesterol (TC), calcium (Ca), and creatine kinase isoenzyme (CK-MB) in group adults were significantly lower than those in groups cubs and sub-adults. The difference is that the values of total bilirubin (T-bil), total egg white (TP), globulin (GLB), and carbon dioxide (CO_2_) in group cubs were significantly lower than those in groups adults and sub-adults.

### 3.5. Correlation Between Gut Microbiota and Blood Indices

**Figure 8:**
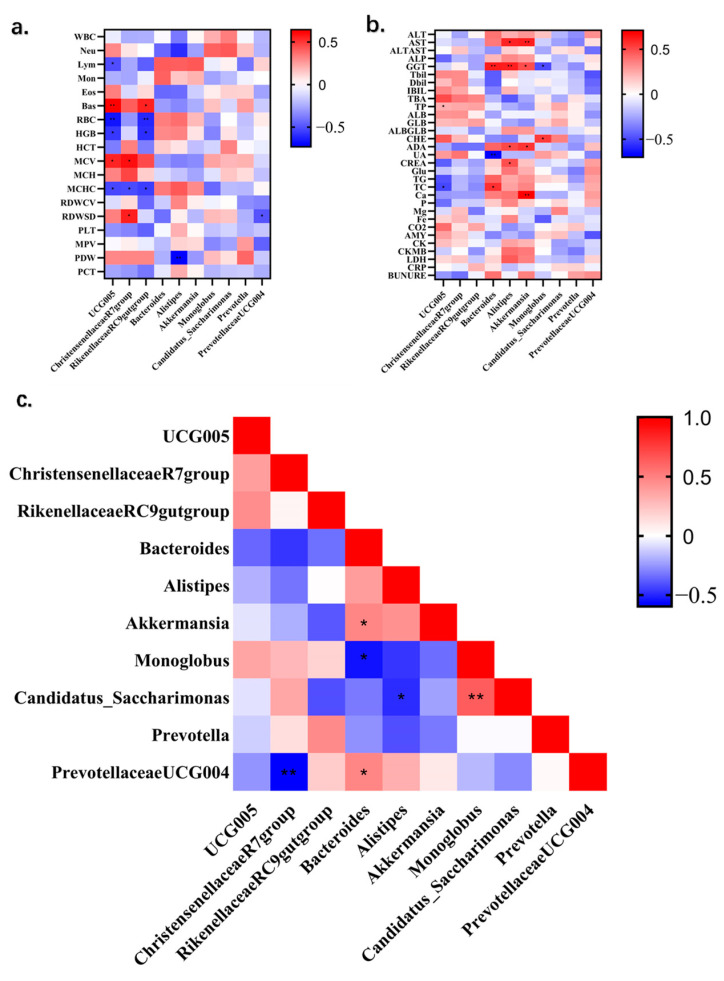
(**a**) The correlation heatmap of microbial abundance in giraffe feces and routine blood values. (**b**) The correlation heatmap of microbial abundance in giraffe feces and serum chemistry. (**c**) The correlation heatmap of microbial abundance between different ages of giraffes. The magnitude of the correlation coefficient was reflected by color intensity. * *p* < 0.05; ** *p* < 0.01.

Through Pearson correlation analysis, we find out the relationship with predominant relative abundance genera of microorganisms at different age stages of giraffes and the blood indices of giraffes. Based on Figure 8, the relative abundance of the *UCG-005* and *Rikenellaceae_RC9_gut groups* at the genus level had a significantly negative correlation with the values of RBC, HGB, and MCHC but were positively correlated with the value of Bas. Additionally, the abundance of the *Christensenellaceae_R7_group* was significantly positive to the values of MCV and RDWSD but negative to the value of MCHC. Meanwhile, there were some other combinations that had a significantly negative correlation between gut microbiota and hematological index, such as *UCG-005* and Lym, *Alistipes* and PDW, and *Prevotellaceae_UCG-004* and RDWSD. Last but not least, the abundance of *UCG-005* was significantly positive with the value of MCV. According to Figure 8, there were many significantly correlations between the predominant relative abundance genera of microorganisms at different age stages of giraffes and the serum biochemistry of giraffes. Among that, the values of AST and ADA were significantly positive with the abundance of *Alistipes* and *Akkermansia*. *Alistipes* was significantly positive to CREA, and *Monoglobus* and *Akkermansia* were also significantly positive to Ca. On the contrary, *Monoglobus* was significantly negative to GGT, and *Bacteroides* was significantly negative to UA. Moreover, the value of GGT was significantly positive to the abundance of *Bacteroides, Alistipes*, and *Akkermansia*. The value of TC was significantly negative to the abundance of *UCG-005* but positive to *Bacteroides*.

### 3.6. Correlation of Gut Microbiota Between Different Age Stages of Giraffes

There was a significantly positive correlation between the abundance of *Bacteroides* and *Akkermansia, Bacteroides* and *Prevotellaceae_UCG-004*, and *Monoglobus* and *Candidatus_Saccharimonas* in each age group of giraffes at the genus level, according to Figure 8. The correlation was different with the abundance of *Christensenellaceae_R7_group* and *Prevotellaceae_UCG-004, Bacteroides* and *Monoglobus*, and *Alistipes* and *Candidatus_Saccharimonas*, which had a significantly negative correlation during every age group of giraffes in this study.

**Figure 9:**
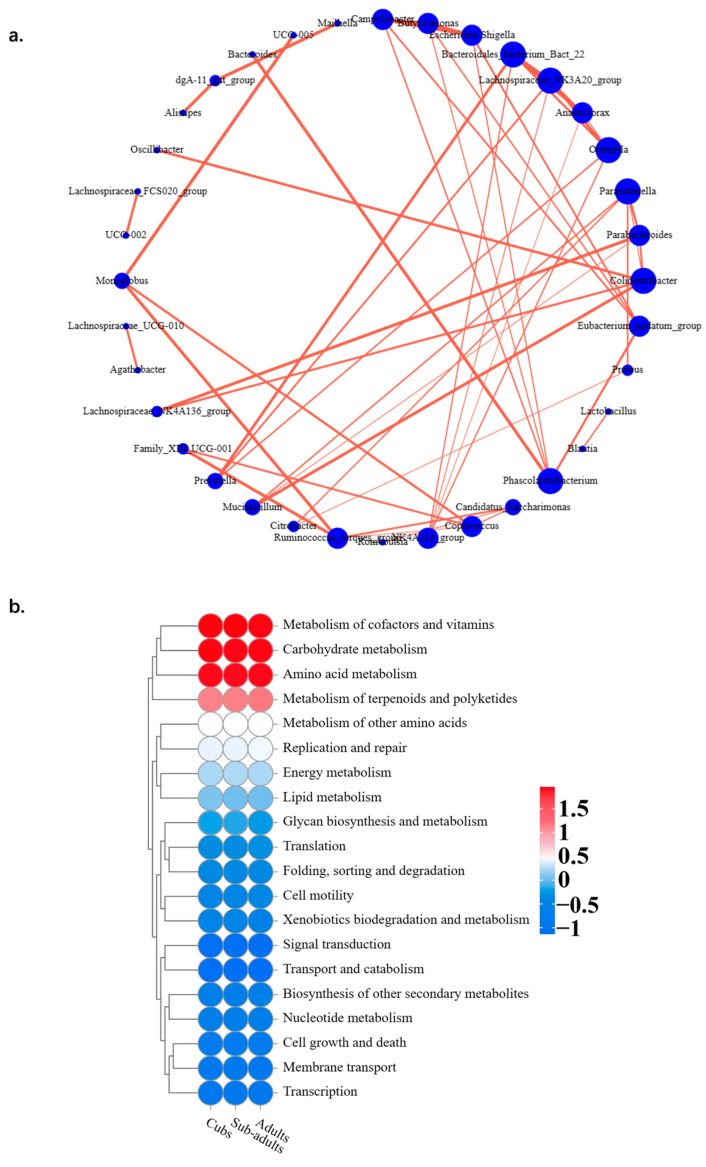
(**a**) Network analysis revealing potential correlation between different bacterial genera. Sizes of circles represent bacterial genera abundance. Correlative strength between both bacteria genera can be evaluated by thickness of the lines. (**b**) Predicted functional distribution of the intestinal microbiota in captive giraffes. The magnitude of the correlation coefficient was reflected by color intensity.

Using the KEGG database, PICRUSt2 software was applied to predict the microbial function in the intestines of giraffes. According to Figure 9, the main functions of intestinal microbiota in giraffes are the metabolism of cofactors and vitamins, carbohydrate metabolism, amino acid metabolism, and metabolism of terpenoids and polyketides.

## 4. Discussion

The mammalian gut bacterial community was normally affected by species, disease, and diet during development and reached stability at maturity [17,18]. In order to find out the variety of intestinal microbes of giraffes in different development stages during their growth and development, we investigated the diversity and structurally characterized abundance of intestinal microbes in giraffes at different developmental stages (cubs, sub-adults, and adults). Given the particularity of giraffes, we selected feces as the research object. This study revealed that Firmicutes, Bacteroidetes, and Proteobacteria were the three most preponderant phyla in the gut microbial community of all samples in groups cubs and sub-adults. Consistent with previous studies on other ruminants, those microorganisms at the phylum level were also abundantly observed in the gut of cattle, yak, and sheep [19,20,21], which indicated the main characteristics of ruminant intestinal microorganisms. Similarly, the three most preponderant phyla of giraffes in this study are the same as the study described by Li et al. [22] Giraffes have a specific gut morphology and metabolism compared to many other animal groups, and it was therefore expected to see a more uniform microbiota composition and less variation between individuals compared to non-ruminants. An herbivorous diet requires a more diverse set of microorganisms to digest efficiently compared to meat-rich diets. The predominant taxa identified in the fecal microbiota across all investigated giraffes regardless of their diet were Firmicutes and Bacteroidetes, which is similar to this study.

However, the situation was different in group adults in that Patescibacteria was the third most preponderant phyla in the gut microbial community at the phylum level of all the samples in group adults. Candidate phyla radiation (CPR), also known as patescibacteria, are unique, extremely small bacteria, and their detection in giraffe feces may be attributed to the local deep well water that the giraffes consume [23]. This finding aligns with results from other giraffes in China [22,24] but contrasts with studies on giraffes in the USA [25], indicating geographical variations in giraffe gut bacterial communities.

Compared to sub-adults and cubs, the abundance of Firmicutes, Patescibacteria, and Spirochaetota in adults was higher, which is consistent with the research studied by Schmidt et al. [25] Conversely, Bacteroidota, Proteobacteria, Actinobacteriota, and Verrucomicrobiota were less abundant in adults. Firmicutes is mainly responsible for the digestion of cellulose, and its higher abundance in the intestinal environment contributes to meet the nutrition and energy requirements of animals during growth and development [26]. Moreover, Firmicutes also contain numerous Gram-positive bacteria, some of which are beneficial, aiding in pathogen resistance and maintaining gut microbiota balance [27], whereas Bacteroidetes have been demonstrated to play a vital role in digesting carbohydrates and proteins and benefit the maturation of the intestinal immune system [28]. Therefore, our findings suggest that, with the increase in animal age, the intestinal microorganisms of giraffes tend to become stable, and the Firmicutes and Bacteroidetes, which are more helpful for digestion, will be more dominant than other flora. On the contrary, some harmful intestinal microorganisms will decrease with the growth of giraffes, such as Proteobacteria and Actinobacteriota. Proteobacteria mainly consist of many Gram-negative bacteria, such as Vibrio cholerae, Helicobacter pylori, Salmonella, and Escherichia coli, which could lead to diarrhea, gastritis, vomiting, gastrointestinal ulcers, and even death, posing a great threat to animal health [29,30]. Similarly, the dramatically increased abundance of Actinobacteria in the gut of goats has been demonstrated to seriously threaten the health of animals [31]. Therefore, we consider that, when giraffes grow from cubs to adults, their intestinal microorganisms tend to be more stable, and beneficial bacteria will be more dominant and harmful bacteria will decrease to improve giraffes’ immunity.

Although the majority of bacteria in different gut segments belong to just a few taxa (such as the Firmicutes, Bacteroidetes, and Proteobacteria), we observed significant bacterial community differences in terms of alpha diversity between giraffes with different ages as well. Alpha diversity of the gut microbial population could be reflected by community abundance (Chao1), diversity index (Shannon and Simpson), and sequencing depth (Good’s coverage) [32]. The phenomenon that the α-diversity of intestinal microbial community will change with the increase in animal age occurs in dairy cows and goats [20,33]. This study found significant differences in alpha diversity among the three age groups, with sub-adults exhibiting higher Chao1 and Shannon indices than cubs but a decrease as sub-adults mature into adults, consistent with Huang et al. [34] We consider that the reason why the α-diversity of intestinal microorganisms in giraffes first increases and then decreases with age is the variation in diet structure. Giraffes have a single food source during the period of cubs, mostly from breastmilk. With the gradual increase in age and the gradual enhancement of digestive ability, giraffes will begin to try more diversified diets, such as eating fresh leaves, which is the reason the α-diversity of sub-adult giraffes is greater than that of cubs. This assumption is similar with the conclusion of Monson [35]. However, the decline of α-diversity in the adult group means intestinal microorganisms tend to be stable. Interestingly, the ASVs and Shannon index of giraffe dung measured in this manuscript are higher than those of other conventional livestock (the average ASVs of giraffe dung measured in this paper is as high as 7177, and the Shannon index is as high as 11 or above). This may be because most of the microbes in giraffe feces have not been annotated, the clustering effect is poor, and they can only be simply classified as the same species without indicating affiliation, thus leading to an overestimation of the Shannon index due to incomplete annotation. We have also reviewed some literature on animal gut microbiota and found cases with a higher number of OTUs and a higher Shannon index, such as the gut microbiota of Chinese miniature horses [36]. Additionally, there are reports on the gut microbiota of 54 mammalian species in zoos, revealing significant differences in gut microbiota among different species. Among them, animals with digestive systems similar to those of giraffes, such as wildebeest and reindeer, have ASVs as high as 7000+, and even giraffes have over 5000 ASVs, which is similar to the results of this manuscript [37]. It is possible that, compared to many other animal groups, ruminants have specific gut morphologies and metabolisms, leading to a more uniform microbial composition and smaller differences between individuals, resulting in better sample homogeneity and a higher Shannon index. Conversely, the gut microbial spectrum of carnivorous animals shows the least diversity among the sampled animals.

The dynamic alternations in gut microbiota between diarrhea and healthy giraffes is also significant, especially Proteobacteria. We observed that the abundance of Proteobacteria and Actinobacteriota in diarrhea giraffes is significantly higher than healthy giraffes at the phylum level but in contrast to the abundance of Spirochaetota and Cyanobacteria, which is consistent with the research results of Li et al. [24].

In previous studies, Proteobacteria and a decrease in Bacteroidetes were proven to be related to the presence of ruminant diarrhea [38]. The decrease in the abundance of treponema giraffes in the diarrhea group is also similar to that of Wu et al. [39]. Species of the phyla Spirochaetota show the potential for hydrolysis of complex polysaccharides in the plant cell wall and toward the production of B-complex vitamins and protein degradation in the rumen [40]. The reduced abundance of Spirochaetota in diarrheal giraffes suggests a decline in digestive capacity. Actinobacteriota, often considered a pathogen causing animal diarrhea, can easily transition to pathogenic interactions [41], so a high variation could be observed at diarrheic cubs for other important phyla, especially for Actinobacteria [31], which is similar with the conclusion of this study. These results indicate a significant alteration in the dominant bacterial phyla of diarrheal giraffes, implying gut microbiota imbalance. Furthermore, the increased prevalence of Proteobacteria could serve as a marker for dysbiosis and a potential diagnostic criterion for disease in the giraffe gut.

Interestingly, the abundance of Cyanobacteria differed significantly between diarrheal and healthy giraffes. Cyanobacteria are less common microbes in ruminant feces than other microorganisms, with an abundance of less than 1%. It was used as a source of functional food due to a potential natural alternative to antibiotics, antiviral, or antifungal therapies [42]. In this study, the relative abundance of Cyanobacteria in diarrhea and healthy giraffes is 0.179% and 0.466%, respectively. Rumen microbial populations are able to gradually change with prolonged, increasing exposure to toxins, thus allowing gradual tolerance of the toxin in ruminants [43]. Meanwhile, the higher abundance of Cyanobacteria in sub-adults compared to cubs indicates that intestinal immunity in giraffes increases with age. A decrease in this probiotic could predispose animals to diarrhea. Consistent with Wu et al. [39], it showed that the gut microbiome of the diarrhea group had a significant decrease in alpha diversity. The alpha diversity in this study in the diarrhea group is lower than the health group, which shows that the cause of diarrhea is the variation of microbial composition, while it had no significant difference.

Importantly, this study also observed a high variation in some bacterial genera between different ages of groups, and this variation may play key roles in the intestinal ecosystem and function. There are six predominant genera at the phylum level that will increase their bacterial abundance with the increase in giraffe age (*UCG-005*, *Christensenellaceae_R-7_group*, *Rikenellaceae_RC9_gut_group*, *Monoglobus*, *Candidatus_Saccharimonas*, and *Prevotella*). *Ruminococcaceae_UCG-005* and *Christensenellaceae_R-7_group* are described as family in the phylum Firmicutes, and they are usually regarded as potential beneficial bacteria, because they participated in the positive regulation of the intestinal environment and are linked to immunomodulation and healthy homeostasis [31,44]. *Candidatus_Saccharimonas* was reported to play an important role in synthesizing enzymes, amino acids, and vitamins and immunomodulation [45]. Those beneficial bacteria can promote good health by enhancing the internal microbial balance [46], which shows that the abundance of intestinal beneficial bacteria in adults is much higher and more stable than that in cubs. What is more, the abundance of *Rikenellaceae_RC9_gut_group* and *Prevotella_UCG-004* in healthy giraffes is significantly higher than that in diarrhea giraffes, which is consistent with the studies described by Li et al. and Xi et al. [22,24] Meanwhile, the abundance of *Monoglobus* in healthy giraffes is significantly higher than diarrhea giraffes as well. All of those three beneficial bacteria that have a significant difference between diarrhea and health were demonstrated to display the characteristics of digesting high carbohydrate food, pectin, and hemicellulose [47,48]. Thus, we suspect that the decrease in bacteria capable of digesting hemicellulose, such as pectin, in the intestinal flora of diarrheal giraffes leads to indigestion and subsequent diarrhea. Moreover, although diarrhea did not significantly differ in gut fungal α-diversity from healthy giraffes, the α-diversity values in diarrheal giraffes were still lower than those in healthy giraffes, including the indices of Chao1, Shannon, and Simpson, further indicating that healthy giraffes generally have a more stable intestinal microbial community.

Patterns of beta diversity were similarly impacted by age variables for the entire community [49]. According to Figure 5 of PCoA, we found that there was obvious separation in β-diversity between different age groups of giraffes and between diarrhea and healthy giraffes in this study, which is similar to the results of previous studies of giraffes [22,24]. The following Adonis analysis results prove this point even more, which shows that it had a significant difference between different age groups and between diarrhea and health (*p* < 0.01). While this result is different from the research written by Pohlin et al. [50], which showed that there was no statistical significance in β-diversity between different ages of Ceratotherium simum.

The hematological parameters are reliable indicators reflecting the physiological and health status of giraffes. Blood components can be influenced by various factors, such as age, parity, days relative to calving, milk production, and season [51], so we assume that there will be differences in the hematological data of giraffes of different ages. To the best our knowledge, this study is the first one to investigate the hematological changes of giraffes of different ages. Cells, such as white blood cells, monocytes, lymphocytes, and granulocytes, are effectors of innate immunity [52]. In this study, we observed that those immunocytes had a higher WBC, monocyte, and lymphocyte counts in sub-adults when compared to cubs but decreased in adults with the age of the giraffes growing up. Among those, the monocyte and lymphocyte counts had a significant difference between the sub-adults and adults. This result is similar to that of other ruminants (buffaloes and cows) [53,54]. Immune cells are gradually increased during animal development, which may be related to their daily breastfeeding, aided by targeting provided by maternal antibodies from the colostrum that cross the gut and circulate in the body. Additionally, there were significant differences (*p* ≤ 0.05) for the red cell hematological constituents between the adults and cubs. In this study, the value of the RBC, HGB, and MCHC in cubs were higher compared to adults, but it was opposite to MCV and MCH, which is consistent with the study of Rusa unicolor and hair goat [55,56]. In general, young animals showed higher mean values for the majority of biochemical values and enzymatic activity as compared to adult animals. These may be attributed to high metabolism in growing animals. However, it was different in some research of dairy cows [54]. Interestingly, a decrease in the absolute number of platelets was observed in this study as the giraffes aged, which was similar to Morita et al. [54], and the PCT also went down with the decrease in PLT.

Serum biochemistry partly reflects the metabolism and health status of animals. This study assumed and found significant differences in the serum biochemical values of giraffes of different ages. Serum TP and ALB reflect the metabolic rate of protein in the body [57] In this study, the values of TP and ALB significantly changed with giraffe growth, indicating that adult giraffes have a stronger immune level compared to cubs and sub-adults. However, the value of Glu significantly decreased with the increasing giraffe age, suggesting that older giraffes have stronger carbohydrate digestion abilities, while younger giraffes are more inclined to consume high-protein diets, such as breastmilk. Previous studies also confirmed the conclusion that Glu will decrease with the increase in the dietary protein level [58]. The concentrations of TG and TC are important indicators for monitoring fat digestion and absorption, which can directly reflect the condition of lipid metabolism in the body [59]. We found that the concentration of TG and TC in cubs is significantly higher than that in adults, which further demonstrates that cubs depend more on a high-protein diet during development, and adults need to mobilize body fat to provide energy for themselves. Alanine aminotransferase (ALT), aspartate aminotransferase (AST), and bilirubin (Bil) are important biomarkers to evaluate hepatocyte damage, which means the existence of oxidative stress [60]. The value of AST in this study had a significant difference between group cubs and adults, but it was the opposite in T-bil, which also had a significant difference between groups cubs and adults. The elevated bilirubin seen in captive giraffes is difficult to interpret [16]. Hyperbilirubinemia is associated with a negative energy balance, which may indicate that the serous atrophy of fat and energy-deficient diets of giraffes could cause per-acute Mortality Syndrome, one of the most common diseases in captive giraffes [61]. While the value of bil in this study is consistent with the International Species Information System (ISIS)17 values for giraffes, which shows that our giraffe is in a healthy condition [16]. CREA is usually a significant marker of kidney biochemistry, which can reflect the functional strength of the kidney [62]. In this study, CREA will go down from cubs to adults, which might indicate that elder giraffes have stronger kidney function to remove creatinine from their blood. However, the CREA of giraffes in this study were commonly higher compared to International Species Information System (ISIS) [16], and it may be reflective of the high-protein diets giraffes are receiving in captivity. It is hypothesized that feeding high-protein diets may be taxing on the kidneys, which work to rid the body of the excess nitrogen, a byproduct of protein digestion, so it is important for breeders to adjust the diet reasonably. Interesting, the control of Ca homeostasis is remarkably precise in vertebrates, whereas the control of P and Mg homeostasis is less stringent. Phosphorus absorption in ruminants is in direct relation to intake, as opposed to Ca, which absorption is regulated based on the Ca status of the animal [63]. According to research, a captive giraffe has lower Ca:P levels than its free-ranging counterparts and that the optimal Ca:P in giraffes is greater than 1 [64]. This is different with captive giraffes in African wild zoos or American zoos, and the ratio of serum calcium and phosphorus measured by them was greater than 1 [16,65]. It is better for ruminants to maintain the ratio of calcium and phosphorus at 2:1, which can keep them in a good calcium and phosphorus metabolism balance. However, the value of Ca:P in this study is smaller than 1.0, and the concentration of P is much greater than other research on giraffes [16]. The bearers of the structural function are the elements that build organs and tissues (Ca and P), and this may explain why the values of Ca and P in cubs are significantly higher than those in adults, because cubs need Ca and P to make sure they can develop their body influentially. What is more, the serum calcium and phosphorus levels of giraffes of different ages are different, and there are many factors affecting the serum biochemical indices of giraffes, such as diet structure, age, and captivity or free range. Koutsos et al. [64] also showed that the level of calcium and phosphorus in giraffes fluctuated greatly under the change in diet structure, and the level of serum calcium and phosphorus in captive giraffes was generally lower than those in free range. Creatine kinase-myocardial band (CK-MB) is widely used in human medicine in the diagnosis of patients with myocardial injury. In Veterinary Medicine, CK-MB has also been used in different ruminant species in the diagnosis of cardiac lesions [66]. We found that CK-MB significantly decreased from cubs to adult giraffes, indicating that the heart develops and the risk of heart injury reduces as giraffes grow. Based on these factors, serum biochemical indices significantly differ in giraffes of different ages, because older giraffes have more mature organs like the heart, liver, and kidneys, making their serum biochemical indices healthier and more stable. Analysis of these blood parameters provides clues to the health status of animals.

To the best of our knowledge, this study is the first to analyze the correlation between fecal microbial flora and hematological indices in giraffes. Gut microbiota dysbiosis leads to the dysregulation of microbiota-related metabolites [67], which could modulate lymphocyte differentiation and cytokine production. Thus, we found a significant negative correlation between Lym counts and *UCG-005*. Recent studies link *Alistipes* to liver cirrhosis [68], consistent with our findings of *Alistipes* correlating with liver condition indices (AST and GGT) in blood biochemistry. Interestingly, *Akkermansia* also correlates with liver indices in this study. However, this strain is considered a potential beneficial bacterium to reverse fatty liver, suggesting that the balance of these two strains indicates good liver function in healthy giraffes. The relationship between Bacteroides and cholesterol in animals has been reported, as it can produce bioactive substances affecting the cholesterol levels [69,70], consistent with the positive correlation between TC in giraffe serum biochemistry and the abundance of Bacteroides in feces in this study.

Originally, we intended to analyze blood from giraffes with diarrhea to better understand the variations in the hematological parameters and their relationship with fecal microorganisms. However, considering the poor physical condition of diarrheal giraffes and the fact that more captive giraffes are desensitized to voluntary blood collection, we did not collect blood from diarrheal giraffes in this study. Therefore, the present results suggest that there are differences in the intestinal microorganisms and hematological indices in giraffes of different ages, and diarrhea leads to an imbalance in intestinal microorganisms, underscoring the importance of exploring practical solutions for regulating the intestinal microbiota of captive giraffes. Additionally, sequencing of the 16S rRNA gene in gut microorganisms is less informative than metagenomics in elucidating microbial functions, and serum metabolomics can reveal the overall changes in giraffe serum metabolism influenced by internal and external factors. It is recommended to use more advanced technologies to study the dynamic changes of intestinal microorganisms and hematology in giraffes.

## 5. Conclusions

In summary, this study investigated the composition and diversity of the intestinal microbiota in captive giraffes and explored the differences in hematological parameters in different age groups of giraffes. It also clarified the effects of diarrhea in giraffes. Firmicutes, Bacteroidota, and Proteobacteria are the predominant bacteria in the intestinal microbiota of captive giraffes. At the genus level, *UCG-005*, *Christensenellaceae_R-7_group,* and *Rikenellaceae_RC9_gut_group* are the predominant genera in the giraffe intestines. Microbial diversity significantly changes with age, with the abundance of the *Rikenellaceae_RC9_gut_group* in adults significantly higher than in sub-adults and cubs (*p* < 0.05).

However, the microbial diversity does not differ between diarrheal and healthy giraffes. The composition and function of the intestinal microbiota significantly differ between diarrheal and healthy giraffes, with the abundance of *Proteobacteria, Spirochaetota*, *Actinobacteriota*, and *Cyanobacteria* significantly different between diarrheal and healthy giraffes (*p* < 0.05). The abundance of *Rikenellaceae_RC9_gut_group*, *Monoglobus*, and *Prevotellaceae_UCG-004* also significantly differs (*p* < 0.05) between diarrheal and healthy giraffes. Lymphocyte (Lym), red blood cell count (RBC), mean red hematocrit (MCV), mean hemoglobin content (MCH), mean hemoglobin concentration (MCHC), platelet number (PLT), and platelet hematocrit (PCT) all significantly differ between different age groups (*p* < 0.05). Similarly, significant differences were observed in some common blood biochemical indices such as AST, TP, CREA, etc. The main functions of the intestinal microbiota in giraffes are the metabolism of cofactors and vitamins, carbohydrate metabolism, amino acid metabolism, and the metabolism of terpenoids and polyketides. There is a significant correlation between the intestinal microbiome composition and hematological parameters in giraffes.

## Figures and Tables

**Table 1:** Routine blood test indices of giraffes at different developmental stages.

Items	Units	Cubs	Sub-Adults	Adults	SEM	*p*-Value
WBC	10 × 10^9^/L	14.33	15.80	13.75	0.569	0.317
Neu#	10 × 10^9^/L	9.376	7.58	10.3983	0.623	0.159
Lym#	10 × 10^9^/L	3.856 ^a^	4.225 ^a^	2.335 ^b^	0.252	0.001
Mon#	10 × 10^9^/L	0.904 ^b^	3.168 ^a^	0.745 ^b^	0.290	0.004
Eos#	10 × 10^9^/L	0.184	0.122	0.242	0.029	0.234
Bas#	10 × 10^9^/L	0.01	0.0133	0.267	0.00371	0.152
RBC	10 × 10^12^/L	9.93 ^a^	9.462 ^a^	8.432 ^b^	0.203	0.002
HGB	g/L	131.4	129.0	119.0	2.664	0.127
HCT	%	31.58	32.07	31.75	0.418	0.904
MCV	fL	31.76 ^b^	33.9 ^b^	37.75 ^a^	0.701	0.000
MCH	pg	13.22 ^b^	13.63 ^ab^	14.13 ^a^	0.133	0.011
MCHC	g/L	415.8	402.3	374.5	5.35	0.001
RDW-CV	%	18.675	18.267	17.25	0.273	0.086
RDW-SD	fL	24.36	23.117	25.167	0.777	0.567
PLT	10 × 10^9^/L	389.4 ^a^	243.667 ^b^	199.833 ^b^	24.40	0.001
MPV	fL	5.900	5.517	5.283	0.110	0.205
PDW	fL	7.62	8.05	8.82	0.296	0.267
PCT	%	0.233 ^a^	0.135 ^ab^	0.104 ^b^	0.167	0.004

SEM = standard error of the mean (n = 6); (a, b) Means within rows with different superscript letters differ at *p* < 0.05. WBC: white blood cell count; Neu#: neutrophil; Lym#: lymphocyte (Lym); Mon#: monocyte; Eos#: eosinophil; Bas#: basophils; RBC: red blood cell count; HGB: hemoglobin concentration; HCT: hematocrit; MCV: mean red hematocrit; MCH: mean hemoglobin content; MCHC: mean hemoglobin concentration; RDW-CV: red cell distribution width-coefficient of variation; RDW-SD: red cell distribution width-standard deviation; PLT: platelet number; MPV: mean platelet volume; PDW: platelet distribution width; PCT: platelet hematocrit.

**Table 2:** Blood biochemical indices of giraffes at different developmental stages.

Items	Units	Cubs	Sub-Adults	Adults	SEM	*p*-Value
ALT	U/L	11.16	14.217	11.833	0.800	0.275
AST	U/L	72.66 ^ab^	80.133 ^a^	64.55 ^b^	2.687	0.039
AST/ALT	-	6.796	5.768	5.725	0.319	0.346
ALP	U/L	612.14 ^a^	523.95 ^a^	230.683 ^b^	46.777	0.000
GGT	U/L	20.06	24.567	19.767	1.294	0.236
T-bil	μmol/L	0.575	0.833	1.967	0.217	0.069
D-bil	μmol/L	1.32	0.75	0.75	0.158	0.275
TBA	μmol/L	30.24	30.55	37.483	3.079	0.577
TP	g/L	66.82 ^b^	83.383 ^a^	88.767 ^a^	2.459	0.004
ALB	g/L	21.62	22.367	23.317	0.334	0.119
GLB	g/L	45.188 ^b^	61.028 ^a^	65.462 ^a^	2.325	0.005
ALB/GLB	-	0.482 ^a^	0.367 ^b^	0.36 ^b^	0.017	0.001
CHE	U/L	58.00	65.167	75.00	3.779	0.197
ADA	U/L	12.54 ^a^	11.9 ^a^	7.00 ^b^	0.793	0.001
CREA	μmol/L	258.52 ^a^	236.12 ^ab^	190.75 ^b^	11.683	0.044
Glu	mmol/L	5.908 ^a^	3.835 ^b^	3.112 ^b^	0.424	0.013
TG	mmol/L	0.533 ^a^	0.510 ^a^	0.213 ^b^	0.049	0.024
TC	mmol/L	2.132 ^ab^	3.268 ^b^	0.683 ^a^	0.324	0.010
Ca	mmol/L	2.388 ^a^	2.24 ^ab^	2.053 ^b^	0.570	0.049
P	mmol/L	4.624	3.982	3.145	0.362	0.270
Mg	mmol/L	0.961	0.825	0.991	0.036	0.111
Fe	μmol/L	20.640	17.267	21.633	1.301	0.362
CO_2_	mmol/L	20.240 ^ab^	21.483 ^b^	26.367 ^a^	0.927	0.007
AMY	U/L	3.060	3.483	5.383	0.486	0.108
CK	U/L	207.08	213.533	165.883	21.566	0.638
CK-MB	U/L	92.82 ^a^	81.183 ^ab^	61.083 ^b^	4.839	0.015
LDH	U/L	557.92	506.667	474.65	24.091	0.407
CRP	mg/L	0.93	1.792	1.912	0.146	0.052
UREA	mmol/L	6.902	8.132	6.748	0.300	0.104

SEM = standard error of the mean (n = 6); (a, b) means within rows with different superscript letters differ at *p* < 0.05. ALT: alanine aminotransferase activity; AST: aspartate aminotransferase; ALP: alkaline phosphatase; GGT: glutamyl transferase activity; T-bil: total bilirubin; D-bil: direct bilirubin; TBA: total bile acid; TP: total protein; ALB: albumin; GLB: globulin; CHE: choline esterase; ADA: adenosine deaminase; CREA: creatinine; Glu: blood glucose; TG: triglyceride; TC: total cholesterol; Ca: calcium; P: inorganic phosphorus; Mg: magnesium; Fe: serum iron; CO_2_: Carbondioxide; AMY: Pancreatic amylase activity; CK: Creatine kinase activity; CK-MB: creatine kinase isoenzyme; LDH: lactic acid dehydrogenase activity; CRP: C-reactive protein; UREA: urea.

## Data Availability

Data are contained within the article or Supplementary Materials.

## Supplementary Materials

The following supporting information can be downloaded at https://www.mdpi.com/article/10.3390/ani14233379/s1: Table S1. The bacterial community composition at the phylum level between different ages of giraffes. Table S2. The bacterial community composition at the phylum level in diarrhea and healthy giraffes. Table S3. The bacterial community composition at the genus level between different ages of giraffes. Table S4. The bacterial community composition at the genus level in diarrhea and healthy giraffes. Table S5. Intestinal microbial of giraffes on α-diversity at different developmental stages. Table S6. Intestinal microbial of diarrhea giraffes on α-diversity. Table S7. Correlation between different bacteria at the genus and routine blood values. Table S8. Correlation between different bacteria at the genus and serum chemistry. Table S9. Correlation of microbial abundance between different ages of giraffes. Table S10. Food intake of giraffes. Table S11. Basic information of experimental giraffes.
