# Supplementary material for: Dynamic Changes in Intestinal Microorganisms and Hematological Indices in Giraffes of Different Ages, and the Effect of Diarrhea on Intestinal Microbiota
Source: Animals (Basel). 2024 Nov 24;14(23):3379. doi: 10.3390/ani14233379 (PMC11640153; doi:10.3390/ani14233379)
Supplement: Supplementary file 1 [file animals-14-03379-s001.zip › animals-3277843-supplementary.pdf]

Table S1. The bacterial community composition at the phylum level between different age of giraffes

Bacteria	Cubs	Sub-adults	Adults	SEM	*P*-value
Firmicutes	54.122^{ab}	51.189^{b}	59.115^{a}	1.282	<0.05
Bacteroidota	30.102	35.08	28.55	1.417	0.145
Proteobacteria	3.376	1.088	0.358	0.69	0.181
Verrucomicrobiota	2.667	2.258	1.083	0.328	0.119
Patescibacteria	1.416	1.067	3.247	0.814	0.643
Spirochaetota	0.592	0.827	1.504	0.208	0.288
Planctomycetota	0.291	0.263	0.157	0.049	0.531
Desulfobacterota	0.662^{ab}	0.753^{a}	0.464^{b}	0.053	0.057
Actinobacteriota	0.272	0.192	0.132	0.031	0.191
Cyanobacteria	0.405^{ab}	0.572^{a}	0.353^{b}	0.038	<0.05

Table S2. The bacterial community composition at the phylum level in diarrhea and healthy giraffes

Bacteria	Diarrhea	Health	SEM	*P*-value
Firmicutes	55.069	54.496	1.998	0.749
Bacteroidota	24.232	30.963	2.824	0.200
Proteobacteria	9.888	0.861	2.155	<0.01
Verrucomicrobiota	2.052	1.989	0.483	0.522
Patescibacteria	0.924	3.487	1.186	0.150
Spirochaetota	0.289	0.870	0.128	<0.01
Planctomycetota	0.296	0.158	0.051	0.337
Desulfobacterota	0.612	0.673	0.110	0.749
Actinobacteriota	0.330	0.150	0.038	<0.05
Cyanobacteria	0.179	0.466	0.058	<0.01

Table S3. The bacterial community composition at the genus level between different age of giraffes

Bacteria	Cubs	Sub-adults	Adults	SEM	*P*-value
UCG-005	9.164	9.568	10.753	0.349	0.156
Christensenellaceae_R-7_group	5.569	5.081	7.526	0.530	0.149
Rikenellaceae_RC9_gut_group	5.388^{b}	6.777^{ab}	7.518^{a}	0.381	0.058
Bacteroides	6.874	6.568	4.516	0.642	0.129
Alistipes	3.137	2.644	2.490	0.188	0.360
Akkermansia	2.481	2.054	0.957	0.323	0.138
Monoglobus	1.496	1.762	1.916	0.109	0.297
Candidatus_Saccharimonas	1.380	1.032	3.218	0.810	0.639
Prevotella	0.427	0.461	0.559	0.055	0.620
Prevotellaceae_UCG-004	1.397	1.433	1.249	0.179	0.916

Table S4. The bacterial community composition at the genus level in diarrhea and healthy giraffes

Bacteria	Diarrhea	Health	SEM	*P*-value
UCG-005	8.601	9.786	0.579	0.337
Christensenellaceae_R-7_group	6.319	6.094	0.533	0.522

Rikenellaceae_RC9_gut_group	3.688	6.419	0.689	<0.05
Bacteroides	3.535	5.680	0.773	0.078
Alistipes	1.350	2.403	0.289	0.078
Akkermansia	1.762	1.828	0.482	0.873
Monoglobus	1.097	1.909	0.178	<0.01
Candidatus_Saccharimonas	0.891	3.451	1.181	0.150
Prevotella	2.027	0.444	0.857	0.522
Prevotellaceae_UCG-004	0.359	1.260	0.190	<0.05

Table S5. Intestinal microbial of giraffe on α-diversity at different developmental stages

Items	Cubs	Sub-adults	Adults	SEM	*P*-value
Goods-coverage	1	1	1	-	-
Chao 1	6303^{b}	8044^{a}	7184.833^{ab}	225.725	<0.01
Shannon	11.132^{b}	11.588^{a}	11.425^{a}	0.064	<0.01
Simpson	0.998556^{b}	0.99899^{ab}	0.99903^{a}	0.0001	<0.05
Pielou	0.8826	0.8934	0.8921	0.0024	0.135

Table S6. Intestinal microbial of diarrhea giraffe on α-diversity

Items	Diarrhea	Health	SEM	*P*-value
Goods-coverage	1	1	-	-
Chao 1	7250.333	7667.833	237.975	0.262
Shannon	11.091	11.517	0.172	0.200
Simpson	0.99513	0.99901	0.00157	0.150
Pielou	0.86500	0.89286	0.01076	0.200

Table S7. Correlation between different bacteria at the genus and routine blood values

	**UCG005**	**ChristensenellaceaeR7group**	**RikenellaceaeRC9gutgroup**	**Bacteroides**	**Alistipes**	**Akkermansia**	**Monoglobus**	**Candidatus_Saccharimonas**	**Prevotella**	**PrevotellaceaeUCG004**
**WBC**	-0.056	-0.245	-0.233	-0.020	-0.391	0.021	0.208	0.329	-0.028	-0.211
**Neu**	0.300	0.066	0.003	-0.438	**-.601***	-0.280	0.399	0.420	0.158	-0.226
**Lym**	**-.510***	-0.259	-0.464	0.411	0.403	0.420	-0.056	0.034	-0.400	0.118
**Mon**	-0.234	-0.261	-0.052	0.396	0.148	0.197	-0.271	-0.198	-0.042	-0.001
**Eos**	0.325	-0.119	0.097	-0.435	-0.425	-0.148	0.050	0.122	0.114	-0.275
**Bas**	**.642****	0.409	**.544***	-0.287	-0.347	-0.245	0.158	-0.123	0.252	-0.156
**RBC**	**-.659****	-0.307	**-.609****	0.329	0.363	0.178	-0.293	0.002	-0.186	0.056
**HGB**	**-.567***	-0.125	**-.596***	0.301	0.317	0.069	-0.359	0.071	-0.167	-0.019
**HCT**	-0.378	0.322	-0.374	0.132	0.071	-0.192	-0.131	0.318	-0.020	-0.059
**MCV**	**.539***	**.588***	0.452	-0.302	-0.368	-0.337	0.235	0.186	0.195	-0.123
**MCH**	0.313	0.463	0.128	-0.109	-0.178	-0.268	-0.094	0.148	0.092	-0.182
**MCHC**	**-.525***	**-.514***	**-.551***	0.339	0.418	0.297	-0.432	-0.201	-0.212	0.019
**RDW CV**	-0.106	0.098	-0.438	-0.153	0.312	0.047	-0.039	0.007	-0.332	-0.363
**RDW SD**	0.321	**.553***	-0.121	-0.417	0.042	-0.208	0.174	0.150	-0.231	**-.488***

**PLT**	-0.317	-0.357	-0.451	-0.025	0.203	0.014	-0.179	-0.131	-0.223	-0.170
**MPV**	-0.019	0.047	-0.064	-0.199	0.111	0.099	-0.348	-0.273	0.257	-0.453
**PDW**	0.303	0.308	0.308	-0.237	**-.731****	-0.299	0.170	0.083	0.384	-0.159
**PCT**	-0.268	-0.303	-0.394	-0.077	0.212	0.028	-0.220	-0.166	-0.153	-0.244

Table S8. Correlation between different bacteria at the genus and Serum chemistry

	**UCG 005**	**Christensenellaceae R7group**	**RikenellaceaeRC9 gutgroup**	**Bacteroides**	**Alistipes**	**Akkermansia**	**Monoglobus**	**Candidatus_Saccharimonas**	**Prevotella**	**PrevotellaceaeUCG004**
**ALT**	-0.036	-0.257	0.030	0.390	0.230	0.292	0.175	-0.214	-0.313	0.314
**AST**	-0.098	-0.171	-0.207	0.378	**.600***	**.611****	-0.132	-0.266	-0.349	0.090
**ALT/AST**	-0.009	0.167	-0.180	-0.297	0.139	0.059	-0.245	0.085	0.119	-0.396
**ALP**	-0.417	-0.204	-0.254	0.201	0.333	0.281	-0.034	-0.196	-0.082	0.093
**GGT**	-0.125	0.051	-0.255	**.618****	**.610****	**.490***	**-.491***	-0.177	-0.312	0.055
**T-bil**	0.332	0.323	-0.059	-0.451	0.154	-0.085	-0.072	-0.059	-0.197	-0.476
**D-bil**	0.188	0.322	-0.166	-0.447	0.005	-0.072	0.043	-0.076	-0.168	-0.422
**IBIL**	0.335	0.250	0.029	-0.348	0.207	-0.081	-0.122	-0.051	-0.164	-0.385
**TBA**	**.500***	0.384	0.342	-0.207	-0.459	-0.252	0.058	-0.238	0.084	-0.094
**TP**	0.225	0.243	0.259	-0.061	-0.236	-0.300	0.094	0.245	0.049	-0.200

**ALB**	0.300	0.308	0.049	-0.254	-0.038	-0.042	-0.061	0.135	0.041	-0.304
**GLB**	0.195	0.213	0.267	-0.028	-0.245	-0.313	0.109	0.241	0.045	-0.168
**ALB/GLB**	-0.099	-0.085	-0.297	-0.099	0.302	0.289	-0.166	-0.186	-0.093	0.042
**CHE**	0.456	0.202	0.022	-0.329	-0.352	-0.109	**.537***	0.355	-0.260	-0.175
**ADA**	-0.239	-0.395	-0.394	0.430	**.503***	**.556***	-0.126	-0.194	-0.303	0.180
**UA**	0.286	0.389	-0.020	**-.700****	-0.208	-0.210	0.277	0.175	-0.031	-0.340
**CREA**	-0.441	-0.167	-0.267	0.146	**.492***	0.172	-0.129	-0.058	-0.191	0.275
**Glu**	-0.167	-0.083	-0.224	0.107	0.382	0.228	0.013	-0.193	-0.358	0.317
**TG**	-0.477	-0.212	-0.289	0.442	0.243	0.304	0.136	0.093	-0.286	0.266
**TC**	**-.505***	-0.206	-0.322	**.565***	0.265	0.343	-0.018	-0.015	-0.171	0.238
**Ca**	-0.288	-0.270	-0.278	0.363	0.396	**.709****	-0.217	-0.233	-0.046	0.278
**P**	-0.403	-0.138	-0.148	0.151	0.341	0.143	0.013	0.121	-0.035	0.232
**Mg**	-0.111	0.184	-0.376	-0.033	0.044	-0.129	-0.204	0.347	-0.102	-0.165
**Fe**	0.124	0.204	-0.103	-0.093	0.341	-0.104	-0.451	-0.081	-0.142	-0.076
**CO2**	0.408	0.086	0.253	-0.069	-0.310	-0.196	0.015	0.075	0.164	-0.096
**AMY**	0.269	0.149	-0.082	-0.338	-0.208	-0.306	0.129	0.177	-0.179	-0.471
**CK**	0.186	0.109	-0.030	-0.122	0.261	0.207	0.028	-0.276	-0.228	-0.118
**CKMB**	-0.148	-0.010	-0.303	0.051	0.404	0.376	-0.003	-0.263	-0.366	-0.129
**LDH**	0.113	0.159	-0.156	0.127	0.458	0.358	-0.152	-0.191	-0.248	0.130
**CRP**	0.017	-0.063	0.214	0.074	-0.186	-0.083	-0.023	0.116	0.126	-0.041
**BUNURE**	-0.289	-0.392	-0.041	0.397	-0.036	0.355	-0.147	0.012	0.269	0.317

Table S9. Correlation of microbial abundance between different age of giraffes

	UCG 005	Christensenellaceae R7group	RikenellaceaeRC 9gutgroup	Bacteroides	Alistipes	Akkermansia	Monoglobus	Candidatus_Saccharimonas	Prevotella	PrevotellaceaeUCG004
UCG005	1	0.385	0.448	-0.357	-0.185	-0.069	0.360	-0.071	-0.118	-0.255
Christensenellaceae R7group		1	0.043	-0.466	-0.329	-0.194	0.284	0.350	0.131	-.597**
RikenellaceaeRC9 gutgroup			1	-0.341	0.005	-0.392	0.173	-0.412	0.461	0.204
Bacteroides				1	0.392	.477*	-.543*	-0.315	-0.263	.479*
Alistipes					1	0.430	-0.466	-.487*	-0.411	0.323
Akkermansia						1	-0.342	-0.226	-0.319	0.091
Monoglobus							1	.634**	-0.010	-0.169
Candidatus_Saccharimonas								1	-0.014	-0.278
Prevotella									1	0.018
PrevotellaceaeUCG004										1

Table S10. Food intake of giraffes

Composition of some feedstuffs commonly fed to giraffes	***Adults***	***Sub-adults***
Alfalfa hay	*10kg*	*5kg*
Silage alfalfa hay	*2.5kg*	*1kg*
concentrate	*2kg*	*1kg*
Alfalfa pellet feed	*0.2kg*	*0.2kg*
Fresh leaves	*3kg*	*2kg*
Fresh carrots	*1kg*	*0.5kg*
Jujubes/eggs	*0.25kg*	*0.25kg*

Table S11. Basic information of experimental giraffes

Sample number	***Name***	***Date of birth***	***Sex***	***Weight(kg)***	***Group***
B-1	*222*	*15 May 2022*	*Female*	*190*	*Cub*
B-2	*092*	*27 April 2022*	*Male*	*240*	*Cub*
B-3	*303*	*25 August 2022*	*Female*	*216*	*Cub*
B-4	*372*	*30 April 2022*	*Male*	*300*	*Cub*
B-5	*312*	*26 June 2022*	*Female*	*235*	*Cub*
B-6	*212*	*22 July 2022*	*Male*	*141*	*Cub*
C-1	*332*	*9 August 2021*	*Female*	*340*	*Sub-adult*
C-2	*331*	*24 August 2019*	*Female*	*563*	*Sub-adult*
C-3	*261*	*1 May 2019*	*Female*	*584*	*Sub-adult*
C-4	*192*	*12 August 2019*	*Female*	*339*	*Sub-adult*
C-5	*061*	*2 June 2019*	*Female*	*558*	*Sub-adult*
C-6	*311*	*25 June 2019*	*Male*	*528*	*Sub-adult*
D-1	*F33*	*2011-2013*	*Female*	*814*	*Adult*
D-2	*F15*	*2011-2013*	*Female*	*820*	*Adult*
D-3	*F2*	*2011-2013*	*Female*	*818*	*Adult*
D-4	*F20*	*2011-2013*	*Female*	*740*	*Adult*
D-5	*F5*	*2011-2013*	*Female*	*774*	*Adult*
D-6	*F37*	*2011-2013*	*Female*	*720*	*Adult*
